# Nanoaerosols Including Radon Decay Products in Outdoor and Indoor Air at a Suburban Site

**DOI:** 10.1155/2012/510876

**Published:** 2012-02-27

**Authors:** Mateja Smerajec, Janja Vaupotič

**Affiliations:** Department of Environmental Sciences, Radon Center, Jožef Stefan Institute, Jamova cesta 39, 1000 Ljubljana, Slovenia

## Abstract

Nanoaerosols have been monitored inside a kitchen and in the courtyard of a suburban farmhouse. Total number concentration and number size distribution (5–1000 nm) of general aerosol particles, as measured with a Grimm Aerosol SMPS+C 5.400 instrument outdoors, were mainly influenced by solar radiation and use of farming equipment, while, indoors, they were drastically changed by human activity in the kitchen. In contrast, activity concentrations of the short-lived radon decay products ^218^Po, ^214^Pb, and ^214^Bi, both those attached to aerosol particles and those not attached, measured with a Sarad EQF3020-2 device, did not appear to be dependent on these activities, except on opening and closing of the kitchen window. Neither did a large increase in concentration of aerosol particles smaller than 10 or 20 nm, with which the unattached radon products are associated, augment the fraction of the unattached decay products significantly.

## 1. Introduction

Air is an aerosol with suspended particulate matter. The particle size ranges from several nm for molecular clusters to about 100 *μ*m for fog droplets and dust particles. Particles larger than 100 *μ*m cannot remain suspended in air and may not therefore be considered as aerosols [1]. The particle size, structure, and chemical composition of aerosols are of key importance for climate and environmental health and are therefore of great interest to aerosol scientists, atmospheric chemists and physicists, and toxicologists and are of serious concern to the regulatory bodies responsible for public health [2–4].

Particulates are emitted by a number of various human activities. They are released by various industries, such as thermal power plants burning fossil fuel or biomass, incinerators, mineral mining and milling facilities, and others. In urban areas where an important or even major particle source is traffic [3–9], aerosol concentration is an order of magnitude higher than those in suburban or rural areas. Nanoparticles are also produced intentionally [10] to be used as constituents in electronics, medicines, pharmaceuticals, cosmetics, paints, and a variety of other consumers products. Nanotechnology is increasing fast and so is the possibility for the nanoparticles to appear in the air of workplaces and be released into the outdoor atmosphere and subsequently enter living environments [11].

During breathing of air, aerosol particulates are partly deposited on the walls of the respiratory tract. Mathematical simulations have shown that their deposition strongly depends on the particle size [12–15]. Thus, for instance [16], about 90% of the inhaled 1 nm particles are deposited in the nasopharyngeal region and the rest in the tracheobronchial region, with no deposition in the alveolar region. Five nm particles are almost equally deposited in all three regions. On the other hand, half of the 20 nm particles are deposited in the alveolar region and the remaining half equally in the other two regions. Physical translocation and clearance in the respiratory tract are also size dependent. Aerosol particles enter the body also by ingestion and absorption through skin. This uptake is more efficient for smaller particles than for larger ones; nonetheless it is minor in comparison to inhalation. Because the ratio of the numbers of surface versus bulk atoms exponentially increases with reducing size, smaller particles are expected to be chemically and biochemically more reactive, and thus potentially more toxic, than larger ones [16]. It has been now recognised that nanoparticles cause oxidation stress, pulmonary inflammation, and cardiovascular events, although the mechanisms of these detrimental effects are not yet understood entirely [4, 16–18]. Aerosols also have an indirect effect on human health because they serve as a carrier for the uptake of airborne radionuclides by inhalation, as explained below.

Three isotopes of radioactive noble gas radon are created by *α*-transformation of radium within the primordial radioactive decay chains in the earth's crust [19]: ^220^Rn (thoron, half-life *t*_1/2_ = 55.6 s) from ^224^Ra in the ^232^Th chain, ^222^Rn (radon, 3.82 days) from ^226^Ra in the ^238^U chain, and ^219^Rn (actinon, 3.9 s) from ^223^Ra in the ^235^U chain. Due to its recoil energy, a fraction of radon atoms succeed in leaving the mineral grain and thus enter the void space. From there, radon travels through the medium either by diffusion or, more effectively and over longer distances, carried by gas or water [20]. On its way, it accumulates in underground rooms (mines, karst caves, fissures, basements) and eventually enters the atmosphere and appears in the air of living and working environments. Usually only ^222^Rn appears at significant levels in the ambient air because of its very long half-life, as compared with the half-life of  ^220^Rn and especially that of  ^219^Rn. We will deal here with ^222^Rn and will call it hereafter radon or Rn.

Radon (^222^Rn) *α*-transformation is followed by a radioactive chain of its successive short-lived decay products (RnDP): ^218^Po (*α*, 3.05 min) → ^214^Pb (*β* and *γ*, 26.8 min) → ^214^Bi (*β* and *γ*, 19.7 min) → ^214^Po (*α*, 164 *μ*s) [19]. Initially, the products appear mostly as positive ions [21–23], which react with molecules of trace gases and vapours (mostly water) in air, are partly oxidized, and form small charged clusters. Eventually, they become neutralised [22, 24]. These processes are accompanied and followed by attachment of clusters [23, 25–28], both charged and already neutralised, to background atmospheric aerosol particles. According to a review by Porstendörfer and Reineking [22], the activity median diameter (AMD) of the RnDP clusters falls into the range from 0.9 nm to 30 nm, while the activity median aerodynamic diameter (AMAD) of the aerosol particles carrying RnDP attached falls in the range from 50 nm to 500 nm. In a radon chamber containing carrier aerosol, AMD values of 0.82, 0.79, 1.70, and 0.82 nm were obtained for the unattached ^218^Po, ^214^Pb, ^214^Bi, and ^214^Po, respectively, [29]. The border between unattached and attached is not fixed. Thus, for indoor air, RnDP associated with particles smaller than 20 nm, grouped around 5 nm [30], and particles in the 0.5–1.5 nm size range may be considered as unattached RnDP [31]. Measurements in indoor air also showed that within the unattached region of <10 nm, two (with AMD of 0.80 and 4.20 nm) or even three activity size distribution peaks (0.60, 0.85 and 1.25 nm) may appear [32, 33]. In addition, RnDP appeared in the nucleation (attached to particles of 14–40 nm), accumulation (210–310 nm), and coarse modes (3000–5000 nm) [32]. In an intercomparison experiment carried out in a test chamber, the AMD values of the unattached RnDP were found in the range from 0.53 to 1.76 nm, followed by a gap until about 50 nm when the attached RnDP appeared [34].

Total concentration of RnDP in air is reported as equilibrium equivalent activity concentration (*C*_RnDP_^A^, Bq m^−3^), expressed as [19]

(1) $$C_{\text{RnDP}}^{\text{A}}=0.1065{C_{218}^{\text{A}}}_{\text{Po}}+0.515{C_{214}^{\text{A}}}_{\text{Pb}}+0.379{C_{214}^{\text{A}}}_{\text{Bi}},$$

where *C* ^A^ (Bq m^−3^) stands for the individual activity concentrations of ^218^Po, ^214^Pb, and ^214^Bi. Because of its short half time, ^214^Po activity is equal to the activity of ^214^Bi and is therefore already included in the last term of (1). Due to air movement and deposition of RnDP on surfaces, individual activity concentrations of RnDP are always lower than that of radon (*C*_Rn_^A^), and the secular equilibrium between radon and RnDP is never reached in the ambient air, its degree being described by the equilibrium factor defined as [19]

(2) $$F=\frac{C_{\text{RnDP}}^{\text{A}}}{C_{\text{Rn}}^{\text{A}}}.$$

Because on the world average, RnDP contribute about half (radon contribution is minor) to the effective dose that a member of the general public receives from all natural radioactivity [35] and are a major cause of lung cancer, second only to cigarette smoking [36], their levels in living and working environments are of serious social concern, and present a great scientific challenge.

For general purposes of radon dosimetry, the International Commission on Radiological Protection in its Publication 65 recommends a dose conversion factor (*D*_CF-E_) of 4 mSv WLM^−1^ and 5 mSv WLM^−1^ at home and in the workplace, respectively, [37], as the conventional values deduced from results of epidemiological studies. Above, 1 WLM (working-level-month) is the exposure gained by 170 hour breathing air in which potential *α*-energy concentration of RnDP (*E*_*α*RnDP_) is 1.3 × 10^8^ MeV m^−3^. *E*_*α*RnDP_ (MeV m^−3^) is expressed through the activity concentrations (*C* ^A^, Bq m^−3^) of  ^218^Po, ^214^Pb, and ^214^Bi, as [19]

(3) $$E_{\alpha \text{RnDP}}=3690{C_{218}^{\text{A}}}_{\text{Po}}+17830{C_{214}^{\text{A}}}_{\text{Pb}}+113120{C_{214}^{\text{A}}}_{\text{Bi}}.$$

On the other hand, dose conversion factors can be calculated by applying dosimetric models [12–15]. Following this approach, Birchall et al. [38, 39] elaborated the procedure for calculating dose conversion factor (*D*_CF-D_), which is used mainly for research purposes. They showed that the parameter most affecting *D*_CF-D_, and thus the calculated effective dose, is the fraction (*f* ^un^) of the unattached RnDP, defined as [19]

(4) $$f^{\text{un}}=\frac{C_{\text{RnDP}}^{\text{Aun}}}{C_{\text{RnDP}}^{\text{A}}}.$$

Above, *C*_RnDP_^Aun^ is the equilibrium equivalent concentration of the unattached RnDP, obtained if activity concentrations of only the unattached ^218^Po, ^214^Pb, and ^214^Bi are taken into (1). Further, they expressed *D*_CF-D_ based on *f* ^un^ with an empirical formula:

(5) $$D_{\text{CF-D}}=11.35+43f^{\text{un}}.$$

As reviewed by Porstendörfer and Reineking [22], *f* ^un^ differs substantially from environment to environment and place to place, and its value ranges from 0.006 to 0.83. It appears to be inversely proportional to the number concentration of the background aerosol particles [40, 41] and thus to be very low in mines with high aerosol concentration [40] and high in karst caves with very clean air [40–45]. In indoor air, ventilation plays a dominant role in governing *F*, *C*_RnDP_^A^, and *f* ^un^, with latter being significantly lower when air condition is used than in places with electric fans or natural ventilation (opening doors and windows) [46, 47]. Important is also the outside environment as a source of particulate matter, such as proximity of, for example, other buildings, parks, roads, and so forth [47]. The influence of meteorological parameters (temperature, barometric pressure, air humidity, wind speed, and rainfall) [48] is less expressed in air-conditioned places [47, 49].

Therefore, knowledge of the number concentration and size distribution of aerosol particles, to which RnDP are associated, is a prerequisite to better understanding the *f* ^un^ values and their temporal variation in an environment [50]. In the present paper, monitoring of nano-aerosols including radon decay products in outdoor and indoor air at a suburban site is described, and results are presented and commented on.

## 2. Experimental

### 2.1. Measuring Techniques

#### 2.1.1. Radon Decay Products

Individual activity concentrations (*C* ^A^, Bq m^−3^) of ^222^Rn, ^218^Po, ^214^Pb, and^ 214^Bi/^214^Po were measured using the EQF3020-2 device (Sarad, Germany). Air is pumped for 6 minutes at a flow rate of 2.4 dm^3^ min^−1^ over a metal mesh grid on which particles smaller than 5 nm (considered as associated with the unattached RnDP) are separated from those above this size (considered as associated with the attached RnDP), and the two fractions are deposited electrostatically on two separate 150 mm^2^ silicon surface barrier detectors. Gross alpha activity is measured during three consecutive intervals within 110 minutes after the end of pumping, and, applying the Markov method [51, 52], individual activity concentrations of  ^218^Po, ^214^Pb, and^ 214^Bi in the unattached (*C*_218_^Aun^_Po_, *C*_214_^Aun^_Pb_, and *C*_214_^Aun^_Bi_) and attached (*C*_218_^Aatt^_Po_, *C*_214_^Aatt^_Pb_, and *C*_214_^Aatt^_Bi_) fractions are obtained. The device also gives radon activity concentration (*C*_Rn_^A^), equilibrium equivalent activity concentration of RnDP (*C*_RnDP_^A^), equilibrium factor between Rn and RnDP (*F*), fraction of the unattached RnDP (*f* ^un^), and potential *α*-energy concentration of RnDP (*E*_*α*RnDP_), as well as air temperature and relative humidity. 

The activity concentrations (*C* ^A^) of radionuclides are converted into their atom number concentrations (*C* ^N^), by applying the radioactivity law equation [19]:

(6) $$\begin{array}{c} C_{}^{\text{A}}=\lambda \times C_{}^{\text{N}}, \end{array}$$

with *λ* equalling the rate constant of radioactive transformation (*λ* = ln⁡2/*t*_1/2_). The obtained number concentrations of ^218^Po, ^214^Pb, and ^214^Bi atoms (cm^−3^) are denoted by *C*_218_^Nun^_Po_,  *C*_214_^Nun^_Pb_ and *C*_214_^Nun^_Bi_, respectively, for the unattached form, and *C*_218_^Natt^_Po_, *C*_214_^Natt^_Pb_, and *C*_214_^Natt^_Bi_, respectively, for the attached form. Also calculated was the fraction *x* ^un^ of the unattached RnDP, expressed by

(7) $$x^{\text{un}}=\frac{{C_{218}^{\text{Nun}}}_{\text{Po}}+{C_{214}^{\text{Nun}}}_{\text{Pb}}+{C_{214}^{\text{Nun}}}_{\text{Bi}}}{{C_{218}^{\text{Nun}}}_{\text{Po}}+{C_{214}^{\text{Nun}}}_{\text{Pb}}+{C_{214}^{\text{Nun}}}_{\text{Bi}}+{C_{\text{218}}^{\text{Natt}}}_{\text{Po}}+{C_{214}^{\text{Natt}}}_{\text{Pb}}+{C_{214}^{\text{Natt}}}_{\text{Bi}}}.$$

In order to avoid confusion, hereafter, *f* ^un^ will be referred to as the activity fraction of the unattached RnDP, and *x* ^un^ as the number fraction of the unattached RnDP.

#### 2.1.2. General Aerosols

Number concentration and size distribution of aerosol particles were measured with a Grimm Aerosol SMPS+C instrument, Series 5.400 (Germany). Its long Vienna DMA unit is designed for 10–1100 nm and a medium DMA unit, for the 5–350 nm size range. The DMA unit separates charged particles into 44 channels based on their electrical mobility, which depends on the particle size and electrical charge. Particles enter the CPC unit containing a heater saturator in which alcohol vapour molecules condense onto the entering particles, thus causing them to grow into droplets. The droplets are then detected with a laser beam (DLS detection) and counted. The frequency of measurement is one in seven minutes for the long unit and one in four minutes for the medium unit. The instrument detects and analyses all the particles, both carrying and not carrying RnDP (although the contribution of the latter is minimal, as will be seen later) therefore the term background aerosol could be misleading, and general aerosol will be used instead. The instrument gives the total number concentration of general aerosol particles *C*_gen_^N^(tot), the geometric mean of their diameters *d*_GM_, and the number size distribution d*C*_gen_^N^(d)/dln*d*  [53], with *d* being the electrical mobility equivalent particle diameter. Because the instrument is designed for >5 nm sizes, the number size distribution of the unattached RnDP below this size could not be evaluated. Therefore, it was adopted [29, 30, 54] that the attached RnDP in indoor air are associated with particles larger than either 10 nm or 20 nm, and the fraction of the general aerosol particles related to the unattached RnDP was expressed by both *x*_gen_(<10) = *C*_gen_^N^(<10)/*C*_gen_^N^(tot) and *x*_gen_(<20) = *C*_gen_^N^(<20)/*C*_gen_^N^(tot). 

#### 2.1.3. Environmental Parameters

The 1/2 hourly average values of the following environmental parameters for outdoor air were obtained from the Slovenian Environment Agency for the Ljubljana Bežigrad meteorological station, approximately 10 km away from our measurement site: air temperature (*T*), barometric pressure (*P*), relative air humidity (*H*_*rel*⁡_), height of precipitation (*h*_rain_), solar radiation (*R*_*s*_), concentrations of NO_x_ (*C*_NO_*x*__), SO_2_ (*C*_SO_2__), O_3_ (*C*_O_3__), and PM10 (*C*_PM10_) (aerosol particles smaller than 10 *μ*m), and wind velocity (*v*_w_). 

### 2.2. Site Description

**Figure 1:**
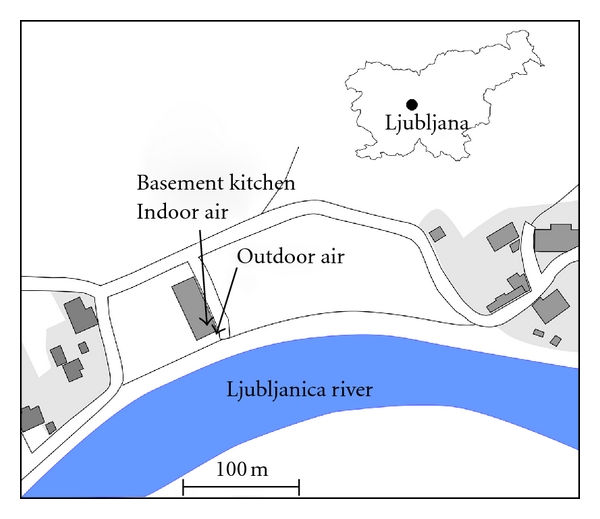
Layout of the measurement site.

Our experiment was carried out in the farm village of Zalog, a suburb of Ljubljana (Slovenia's capital with 370,000 inhabitants). Of the total five farms, the last one at the furthest end of the road was selected, composed of a residential house and several accompanying buildings (Figure 1). They were built in 1987 of concrete and brick. The family lives in the ground floor of the residential house. One person lives temporarily in a small 20 m^2^ flat in the basement, its floor lying 1.2 m below the courtyard level. The flat consists of a kitchen, living room, bathroom, and corridor. A door and a window of the kitchen face the courtyard in front of the house, while the other door connects it to other rooms. Central heating using hot water radiators is based on burning wood. There is no air conditioning. The Ljubljanica river flows at a distance of about 20 m and the nearest neighbour lies about 50 m away. The village is surrounded by fields. Across the river at a distance of about 500 m, the main railway Ljubljana-Maribor runs along a hill covered by forest. The Ljubljana wastewater treatment plant is about 400 m outside the village, and there are some small industrial plants several kilometres away.

Indoor measurements were performed in the kitchen of the basement flat, and outdoor measurements in the courtyard in front of it. Preliminary radon survey had shown elevated radon activity concentration in indoor air, ranging from 300 Bq m^−3^ to 1000 Bq m^−3^, as compared with the national average in winter of 121 Bq m^−3^ for one thousand randomly selected dwellings [55]. With some interruptions because of other measurements, outside measurements were carried out in May 2010 using the long Vienna DMA unit, and in October 2010 using the medium Vienna DMA unit, and indoor measurements were performed from October 2010 to January 2011, also using the medium Vienna DMA unit.

## 3. Results and Discussion

### 3.1. Outdoor Air

**Figure 2:**
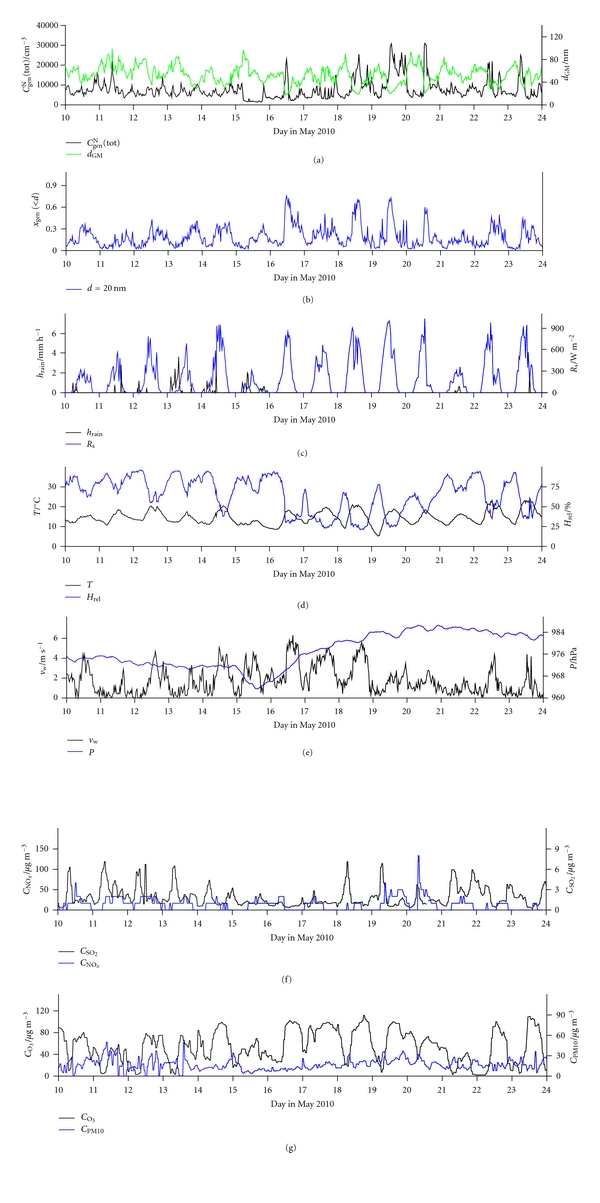
Time run of: (a) the total number concentration of general aerosol particles (*C*_gen_^N^(tot)) and geometric mean of their diameters (*d*_GM_), (b) number fraction of particles smaller than 10 nm (*x*_gen_(<10)) and smaller than 20 nm (*x*_gen_(<20)), (c) precipitation (*h*_rain_) and solar radiation (*R*_*s*_), (d) air temperature (*T*) and air relative humidity (*H*_*rel*⁡_), (e) wind velocity (*v*_w_) and barometric pressure (*P*), (f) concentration of NO_*x*_ (*C*_NO_*x*__) and SO_2_ (*C*_SO_2__), and (g) concentrations of O_3_ (*C*_O_3__) and aerosol particles smaller than 10 *μ*m (*C*_PM10_), in outdoor air in front of the dwelling for the period May 10–23, 2010.

**Figure 3:**
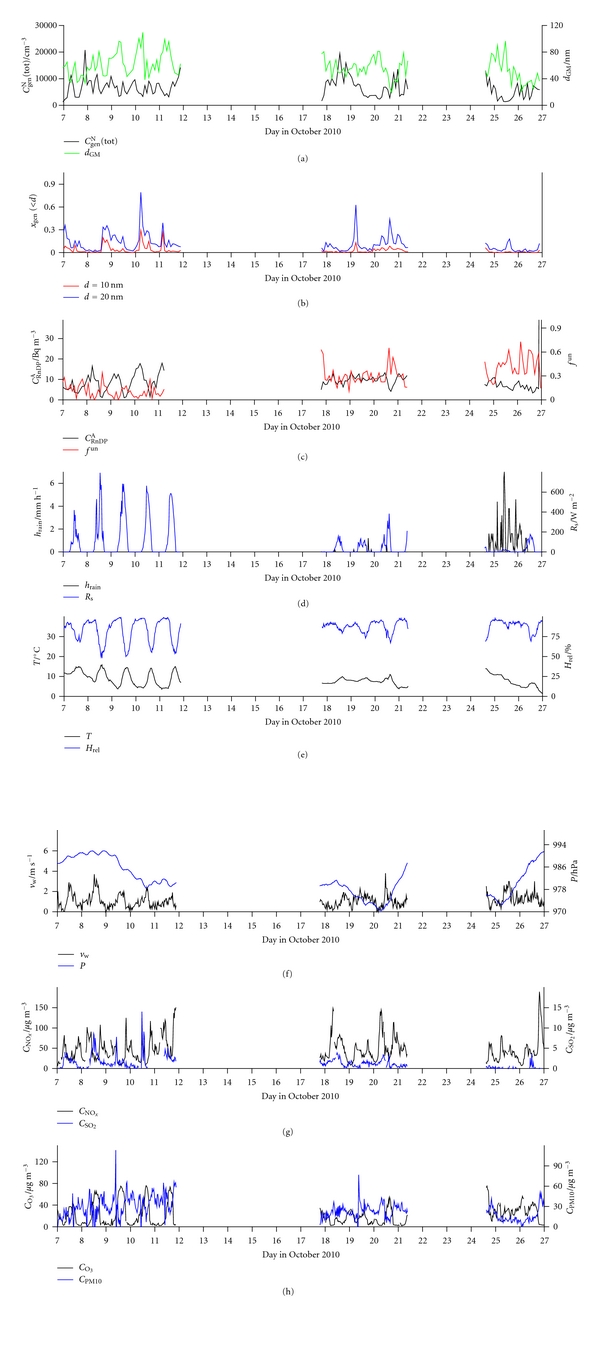
Time run of: (a) the total number concentration of general aerosol particles (*C*_gen_^N^(tot)) and geometric mean of their diameters (*d*_GM_), (b) number fraction of particles smaller than 10 nm (*x*_gen_(<10)) and smaller than 20 nm (*x*_gen_(<20)), (c) precipitation (*h*_rain_) and solar radiation (*R*_*s*_), (d) air temperature (*T*) and air relative humidity (*H*_*rel*⁡_), (e) wind velocity (*v*_w_) and barometric pressure (*P*), (f) concentration of NO_x_ (*C*_NO_*x*__) and SO_2_ (*C*_SO_2__), and (g) concentrations of O_3_ (*C*_O_3__) and aerosol particles smaller than 10 *μ*m (*C*_PM10_), in outdoor air in front of the dwelling for the period October 6–26, 2010.

**Figure 4:**
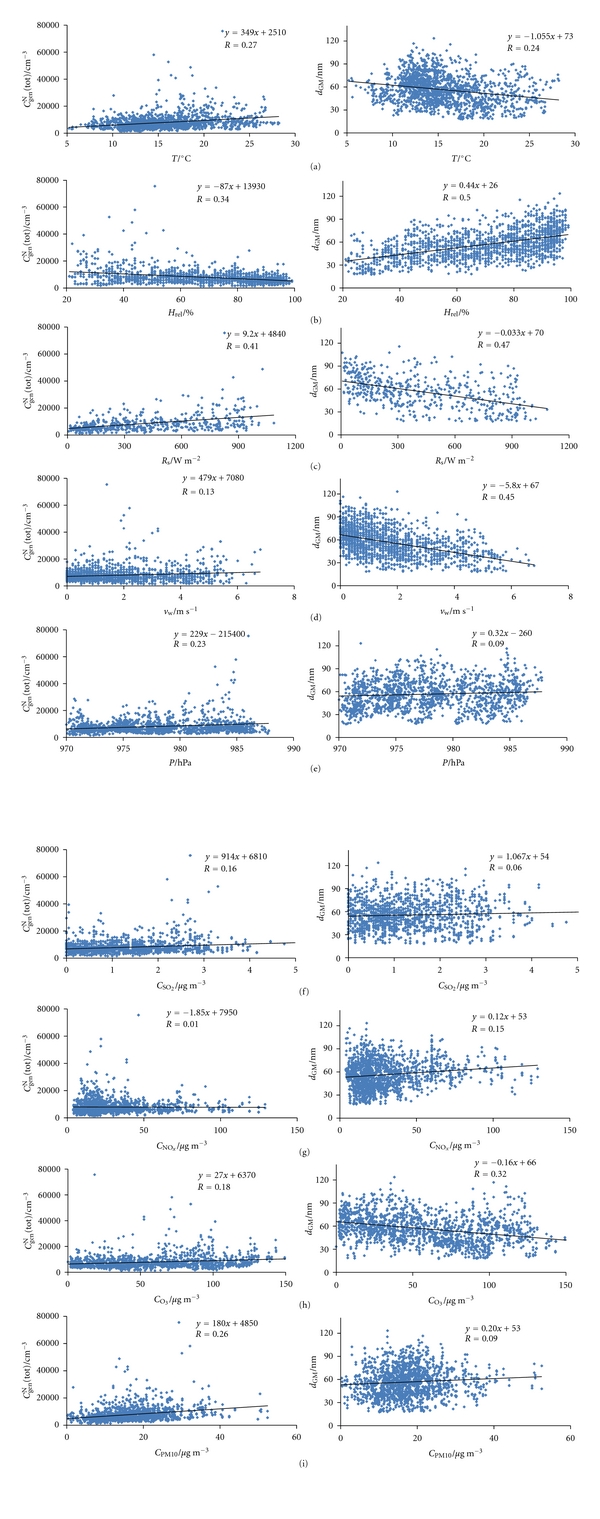
Correlations of the following relationships in outdoor air for the period May 3–June 6, 2010: (a) *C*_gen_^N^(tot)–*T* and *d*_GM_–*T*, (b) *C*_gen_^N^(tot)–*H*_*rel*⁡_ and *d*_GM_–*H*_*rel*⁡_, (c) *C*_gen_^N^(tot)–*R*_*s*_ and *d*_GM_–*R*_s_, (d) *C*_gen_^N^(tot)–*v*_w_ and *d*_GM_–*v*_w_, (e) *C*_gen_^N^(tot)–*P* and *d*_GM_–*P*, (f) *C*_gen_^N^(tot)–*C*_SO_2__ and *d*_GM_–*C*_SO_2__, (g) *C*_gen_^N^(tot)–*C*_NO_*x*__ and *d*_GM_–*C*_NO_*x*__, (h) *C*_gen_^N^(tot)–*C*_O_3__ and *d*_GM_–*C*_O_3__, and (i) *C*_gen_^N^(tot)–*C*_PM10_ and *d*_GM_–*C*_PM10_.

**Figure 5:**
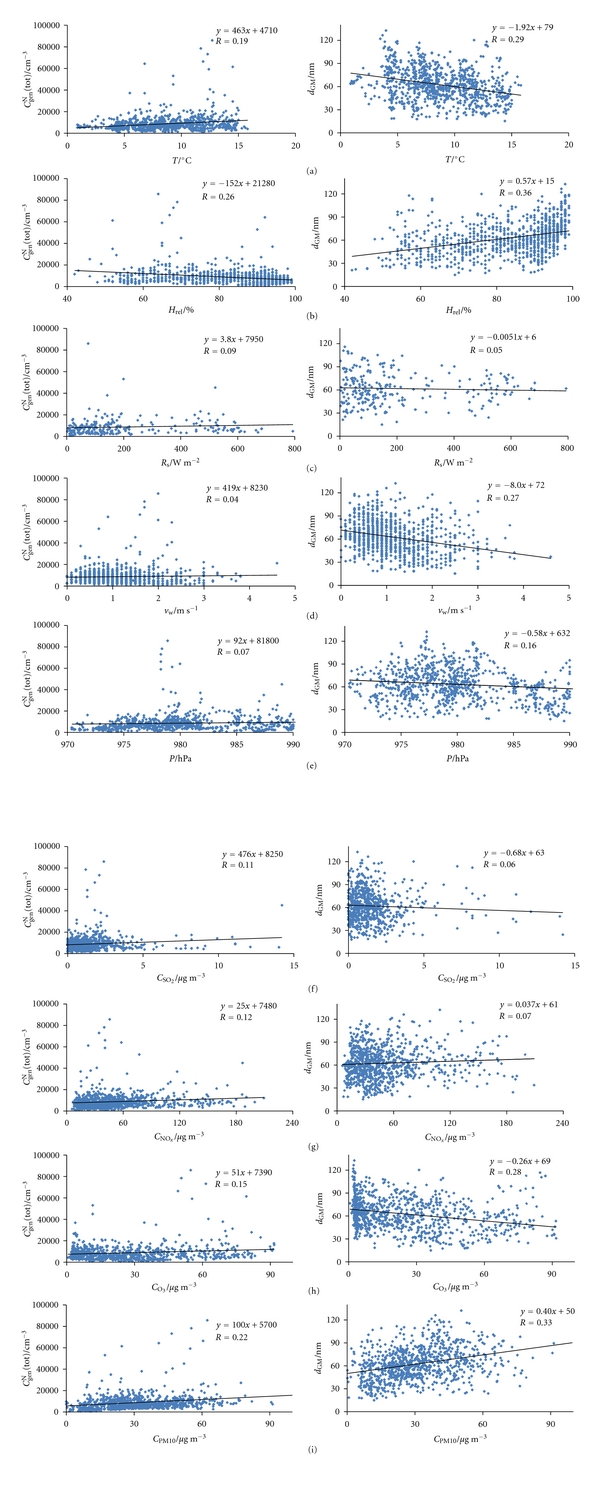
Correlations of the following relationships in outdoor air for the period October 6–26, 2010: (a) *C*_gen_^N^(tot)–*T* and *d*_GM_–*T*, (b) *C*_gen_^N^(tot)–*H*_*rel*⁡_ and *d*_GM_–*H*_*rel*⁡_, (c) *C*_gen_^N^(tot)–*R*_s_ and *d*_GM_–*R*_s_, (d) *C*_gen_^N^(tot)–*v*_w_ and *d*_GM_–*v*_w_, (e) *C*_gen_^N^(tot)–*P* and *d*_GM_–*P*, (f) *C*_gen_^N^(tot)–*C*_SO_2__ and *d*_GM_–*C*_SO_2__, (g) *C*_gen_^N^(tot)–*C*_NO_*x*__ and *d*_GM_–*C*_NO_*x*__, (h) *C*_gen_^N^(tot)–*C*_O_3__ and *d*_GM_–*C*_O_3__, and (i) *C*_gen_^N^(tot)–*C*_PM10_ and *d*_GM_–*C*_PM10_.

Figures 2 and 3 show the total number concentration of general aerosols (*C*_gen_^N^(tot)) and geometric mean of particle diameter (*d*_GM_), together with environmental parameters, for the outdoor measurements carried out in the periods of May 10–23 and October 7–26, respectively. Minimum and maximum values, geometric means and geometric standard deviations are shown in Table 1. Figures 4 and 5 show relationships between *C*_gen_^N^(tot) and *d*_GM_ with the environmental parameters for the May and October measurements, respectively. All correlation coefficients (*R*) are far below 0.50, and hence the dependence of both *C*_gen_^N^(tot) and *d*_GM_ on these parameters is weak. The values of the environmental data taken from the meteorological station 10 km away may not show the actual situation at our measurement site, and the correlations shown are not necessarily realistic. Exceptions could be temperature and solar radiation, whose values may not change markedly in several kilometres. Nonetheless, based on *R* values neither of their effects are evidenced. 

**Figure 6:**
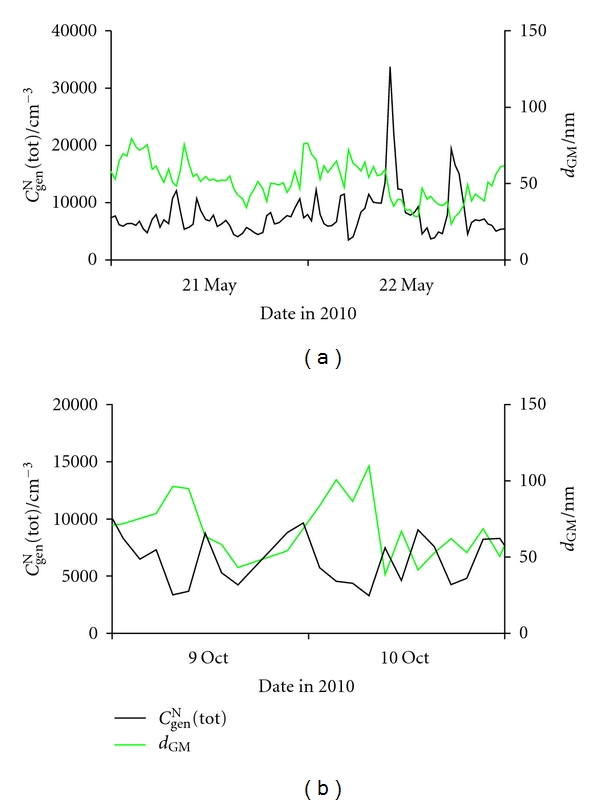
Diurnal variations of the total number concentration of general aerosol (*C*_gen_^N^(tot)) and geometric mean of their diameters (*d*_GM_) in outdoor air for (a) May 21-22 and (b) October 9-10.

**Figure 7:**
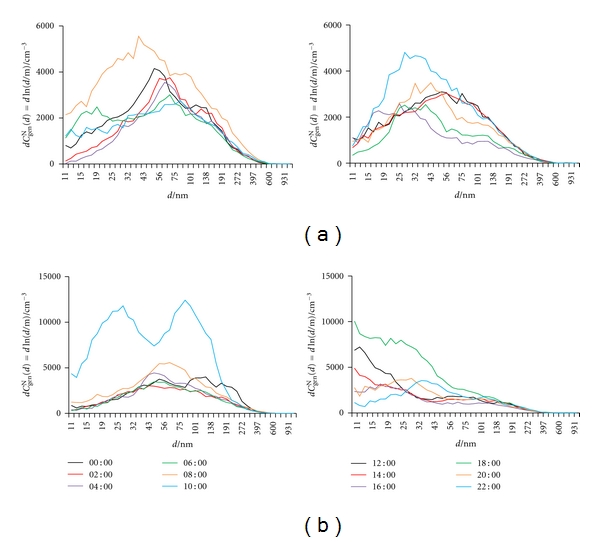
Number particle size distribution (d*C*_gen_^N^(d) = dln(*d*/m)) in outdoor air recorded every two hours on: (a) May 21 and (b) May 22.

**Figure 8:**
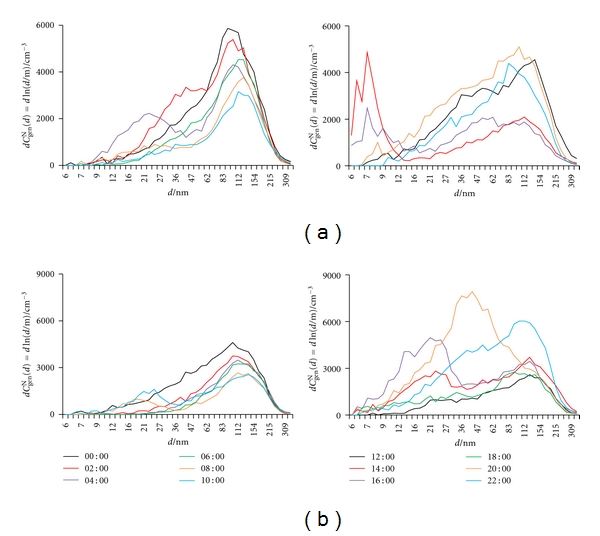
Number particle size distribution (d*C*_gen_^N^(*d*) = dln(*d*/m)) in outdoor air recorded every two hours on: (a) October 9 and (b) October 10.

Figure 6 shows diurnal variations of *C*_gen_^N^(tot) and *d*_GM_ only for two selected days in May and October. For these days, the following correlation coefficients for *R*_*s*_ were obtained: 0.51 for *C*_gen_^N^(tot) and 0.60 for *d*_GM_ in May, and 0.69 for *C*_gen_^N^(tot) and 0.65 for *d*_GM_ in October; hence, solar radiation may be taken into account in data interpretation. In both urban [8, 56, 57] and semirural areas [58], two daily *C*_gen_^N^(tot) peaks were found, one in the morning and the other in late afternoon, coinciding with the traffic rush hours. In our case, no periodicity is seen for May, and changes seem to appear sporadically. Diurnal variation in size distribution differs considerably from day to day, as seen for every two hours in Figure 7. In October, two maxima appear, one at around midnight, and the other at around noon. In this period of the year, farming activities (e.g., running cars, tractors, and other farming equipment) are highest from late morning to early afternoon, with a concomitant strongest solar radiation [56, 59, 60], thus causing the first increase in *C*_gen_^N^(tot), accompanied by a decrease in *d*_GM_. The late afternoon simultaneous rising of both *C*_gen_^N^(tot) and *d*_GM_ is presumably related to farming activities, traffic on a nearby road, and particle growth by coagulation [4, 61]. Decrease in *C*_gen_^N^(tot) after its midnight maximum is ascribed to faster deposition of smaller particles, caused by growing air humidity and appearance of dew [62], thus resulting in increasing *d*_GM_.  Figure 8 shows particle size distribution every two hours on these two days. While larger particles (around 100 nm) prevail in the morning hours, the contribution of smaller ones (smaller than 30 nm) becomes significant when using diesel engines. 

**Figure 9:**
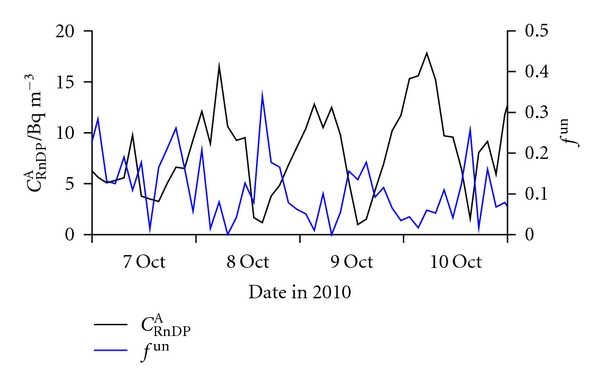
Diurnal variations of the equilibrium equivalent activity concentration of radon decay products (*C*_RnDP_^A^) and fraction of the unattached decay products (*f* ^un^), in outdoor air for the period October 7–10.

Radioactive aerosol particles in outdoor air were monitored only in the periods of October 7–11 (Figure 9) and October 17–26. Diurnal variation in *C*_RnDP_^A^, with maxima overnight and minima at noon, is well pronounced. An expected [40, 41, 63], though only approximate, coincidence of *f* ^un^ minima and *C*_RnDP_^A^ maxima is seen. 

### 3.2. Indoor Air

**Figure 10:**
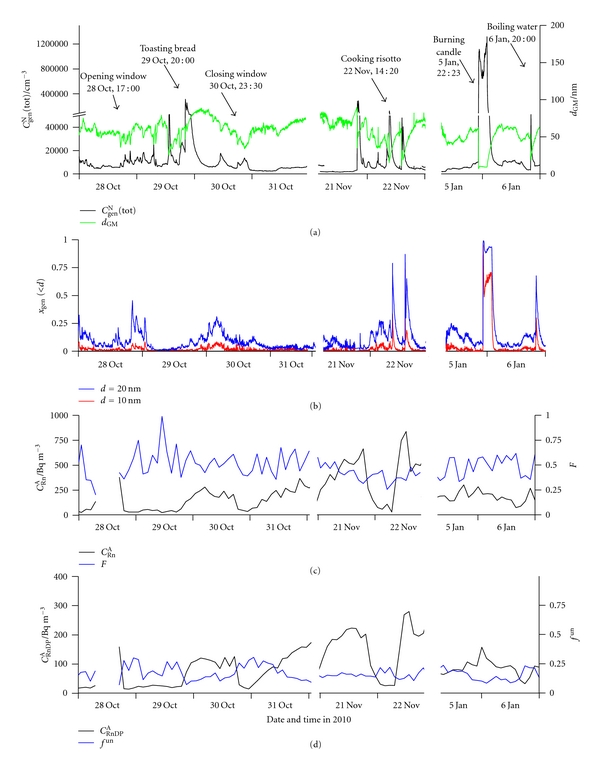
Time run of: (a) total number concentration of general aerosol particles (*C*_gen_^N^(tot)) and geometric mean values of their diameters (*d*_GM_), (b) number fraction of particles smaller than 10 nm (*x*_gen_(<10)) and smaller than 20 nm (*x*_gen_(<20)), (c) activity concentration of radon (*C*_Rn_^A^) and equilibrium factor between Rn and RnDP (*F*), and (d) equilibrium equivalent activity concentration of radon decay products (*C*_RnDP_^A^) and activity fraction of the unattached RnDP (*f* ^un^), in indoor air from October 28 to January 6.

Figure 10 shows the time series of (a) *C*_gen_^N^(tot) and *d*_GM_, (b) *x*_gen_(<10) and *x*_gen_(<20), (c) *C*_Rn_^A^ and *F*, and (d) *C*_RnDP_^A^ and *f* ^un^, in indoor air of the basement kitchen, from October 28, 2010 to January 6, 2011, with some interruptions because of other measurements. During this period, the basement flat was normally inhabited, and all main activities were recorded, such as opening and closing windows, cleaning, preparing food, and other activities presumed to be sources of particulate matter. In the following, only events potentially influenced by human activity will be presented and discussed.

Table 2 shows minimum and maximum values, geometric means, and geometric standard deviations of the parameters monitored in the kitchen of the basement flat from October 28, 2010 to January 6, 2011, only during periods with the door and window closed and without human activity.

#### 3.2.1. Opening the Window

**Figure 11:**
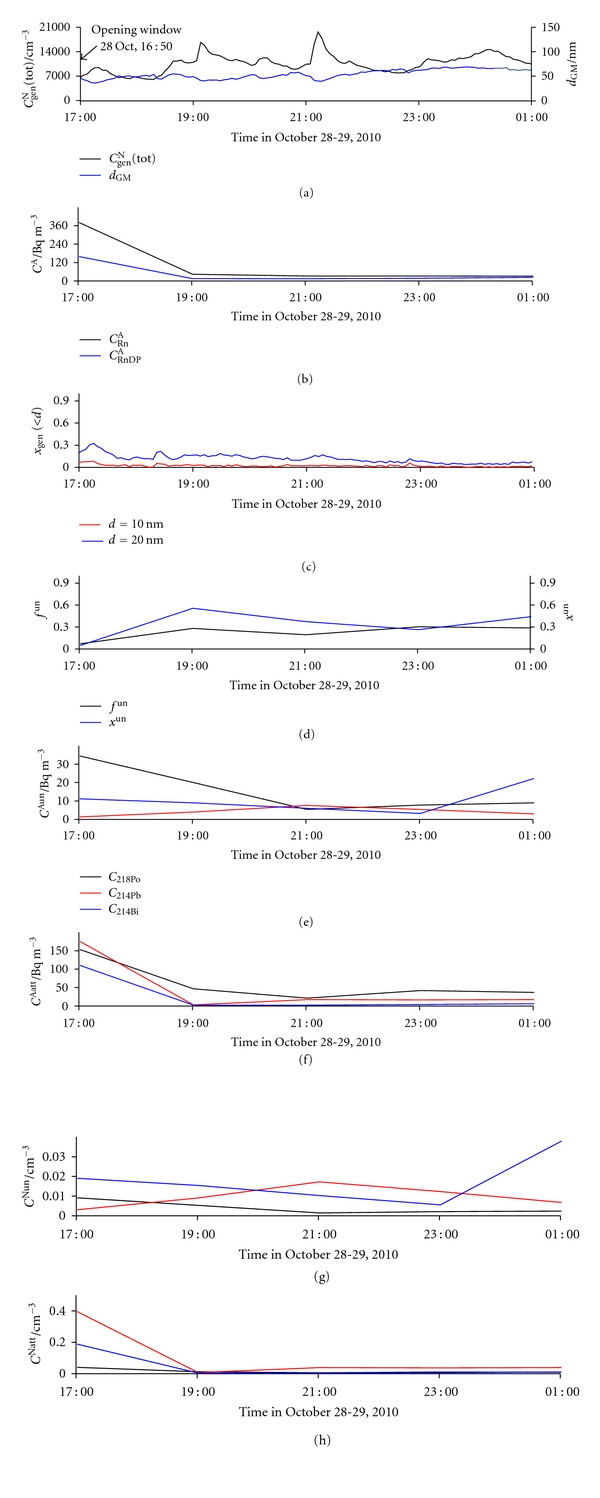
Time run of: (a) total number concentration of general aerosol particles (*C*_gen_^N^(tot)) and geometric mean values of their diameters (*d*_GM_), (b) activity concentration of radon (*C*_Rn_^A^) and equilibrium equivalent activity concentration of radon decay products (*C*_RnDP_^A^), (c) number fraction of particles smaller than 10 nm (*x*_gen_(<10)) and smaller than 20 nm (*x*_gen_(<20)), (d) activity fraction of the unattached RnDP (*f* ^un^) and number fraction of the unattached RnDP (*x* ^un^), (e) activity concentrations of the unattached RnDP atoms (*C*_218_^Aun^_Po_, *C*_214_^Aun^_Pb_, and *C*_214_^Aun^_Bi_), (f) activity concentrations of the attached RnDP atoms (*C*_218_^Aatt^_Po_, *C*_214_^Aatt^_Pb_, and *C*_214_^Aatt^_Bi_), (g) number concentrations of the unattached RnDP atoms (*C*_218_^Nun^_Po_, *C*_214_^Nun^_Pb_, and *C*_214_^Nun^_Bi_), and (h) number concentrations of the attached RnDP atoms (*C*_218_^Natt^_Po_, *C*_214_^Natt^_Pb_, and *C*_214_^Natt^_Bi_), in indoor air for the period October 28-29 (with opening of the window in the kitchen at 16:50 on October 28).

**Figure 12:**
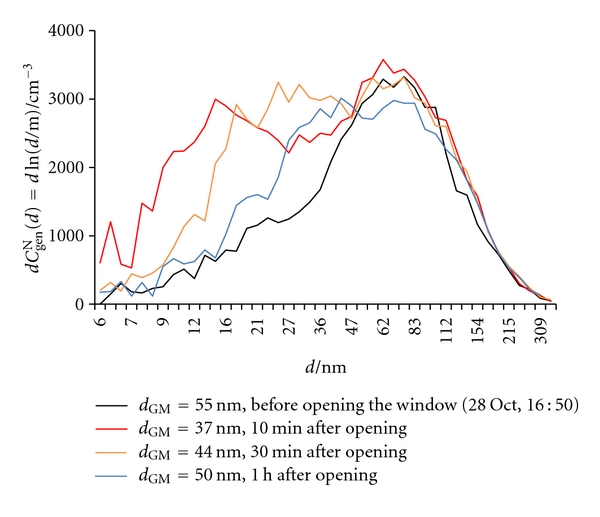
Number size distributions (d*C*_gen_^N^(*d*) = dln(*d*/m)) of general aerosol particles in indoor air before and during opening of the window in the kitchen.

The window in the kitchen was opened at 16:50 on October 28. As expected, both *C*_Rn_^A^ and *C*_RnDP_^A^ decreased suddenly (Figure 11(b)). Because *d*_GM_ in outdoor air is low in the afternoon, the inflow of outdoor air shifted the size distribution towards lower values (Figure 12), thus reducing *d*_GM_ and increasing *x*_gen_(<20) indoors (Figure 11(c)). As a consequence, the probability of RnDP atoms meeting smaller particles is enhanced, and both *f* ^un^ and *x* ^un^ are increased, though not markedly (Figure 11(d)). Nonetheless, the frequency of EQF measurement is too low to follow such abrupt changes, and therefore the calculated *f* ^un^ and *x* ^un^ responses are not necessarily correct. The decrease in number concentrations of all three RnDP in the attached form (Figure 11(h)) is a consequence of the decrease in *C*_RnDP_^A^ (Figure 11(b)).

#### 3.2.2. Closing the Window

**Figure 13:**
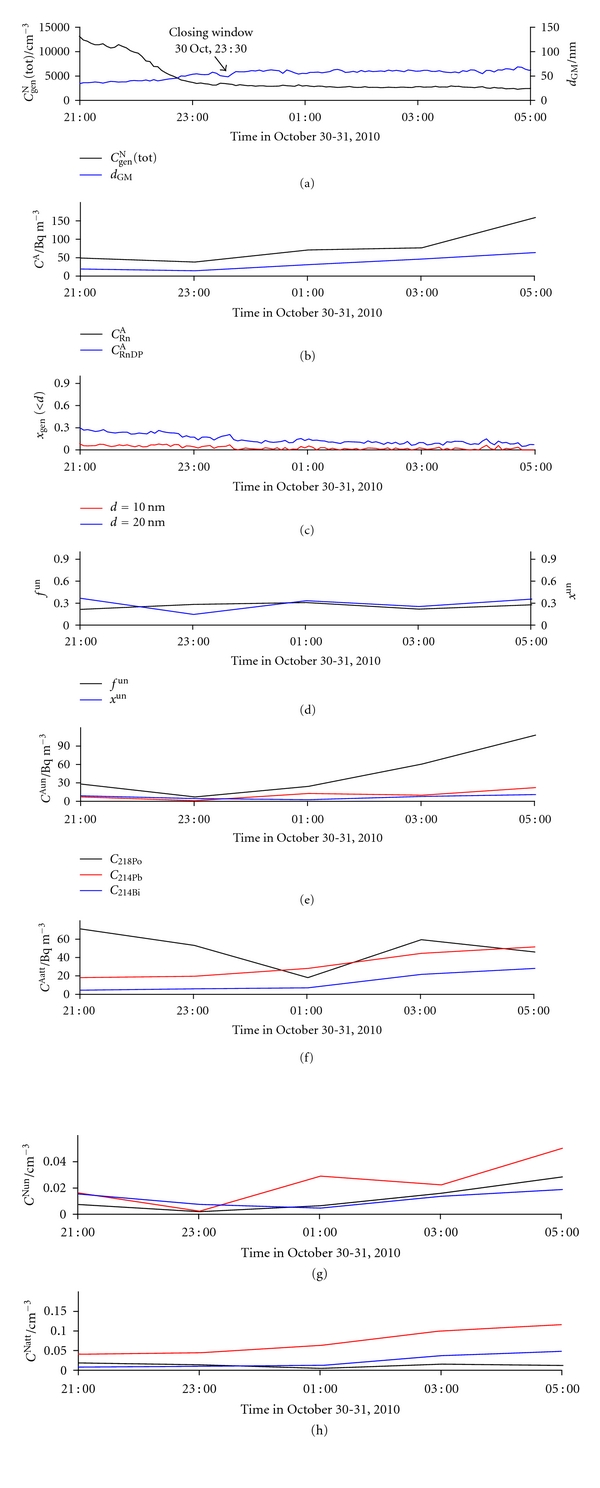
Time run of: (a) total number concentration of general aerosol particles (*C*_gen_^N^(tot)) and geometric mean values of their diameters (*d*_GM_), (b) activity concentration of radon (*C*_Rn_^A^) and equilibrium equivalent activity concentration of radon decay products (*C*_RnDP_^A^), (c) number fraction of particles smaller than 10 nm (*x*_gen_(<10)) and smaller than 20 nm (*x*_gen_(<20)), (d) activity fraction of the unattached RnDP (*f* ^un^) and number fraction of the unattached RnDP (*x* ^un^), (e) activity concentrations of the unattached RnDP atoms (*C*_218_^Aun^_Po_, *C*_214_^Aun^_Pb_, and *C*_214_^Aun^_Bi_), (f) activity concentrations of the attached RnDP atoms (*C*_218_^Aatt^_Po_, *C*_214_^Aatt^_Pb_, and *C*_214_^Aatt^_Bi_), (g) number concentrations of the unattached RnDP atoms (*C*_218_^Nun^_Po_, *C*_214_^Nun^_Pb_, and *C*_214_^Nun^_Bi_), and (h) number concentrations of the attached RnDP atoms (*C*_218_^Natt^_Po_, *C*_214_^Natt^_Pb_, and *C*_214_^Natt^_Bi_), in indoor air for the period October 30-31 (with closing of the window in the kitchen at 23:30 on October 30).

**Figure 14:**
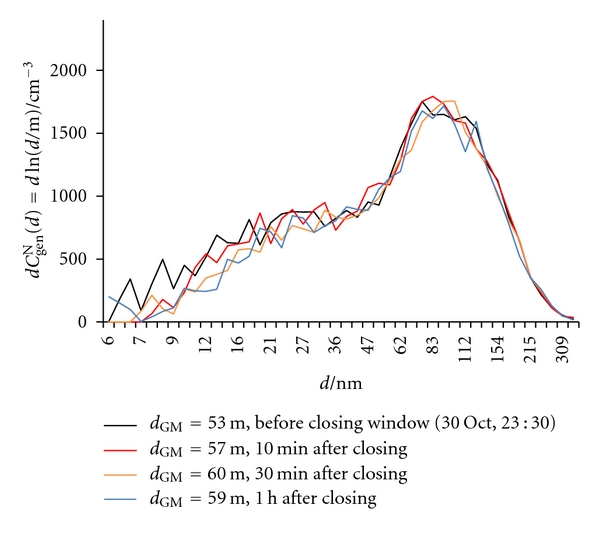
Number size distributions (d*C*_gen_^N^(*d*) = dln(*d*/m)) of general aerosol particles in indoor air before and after closing of the window in the kitchen.

Upon closing the window at 23:30 on October 30, only a slight and slow increase in *d*_GM_ was observed (Figure 13(a)), as shown also in Figure 14. A steady increase in *d*_GM_ and thus decrease in *x*_gen_(<10) and *x*_gen_(<20) follow (Figure 13(c)). Increase in *C*_RnDP_^A^ (Figure 13(b)) is a result of increasing the individual number concentrations of RnDP, both in the unattached and attached forms (Figures 13(g) and 13(h)). Both *f* ^un^ and *x* ^un^ show a slight increase (Figure 13(d)).

#### 3.2.3. Using a Toaster

**Figure 15:**
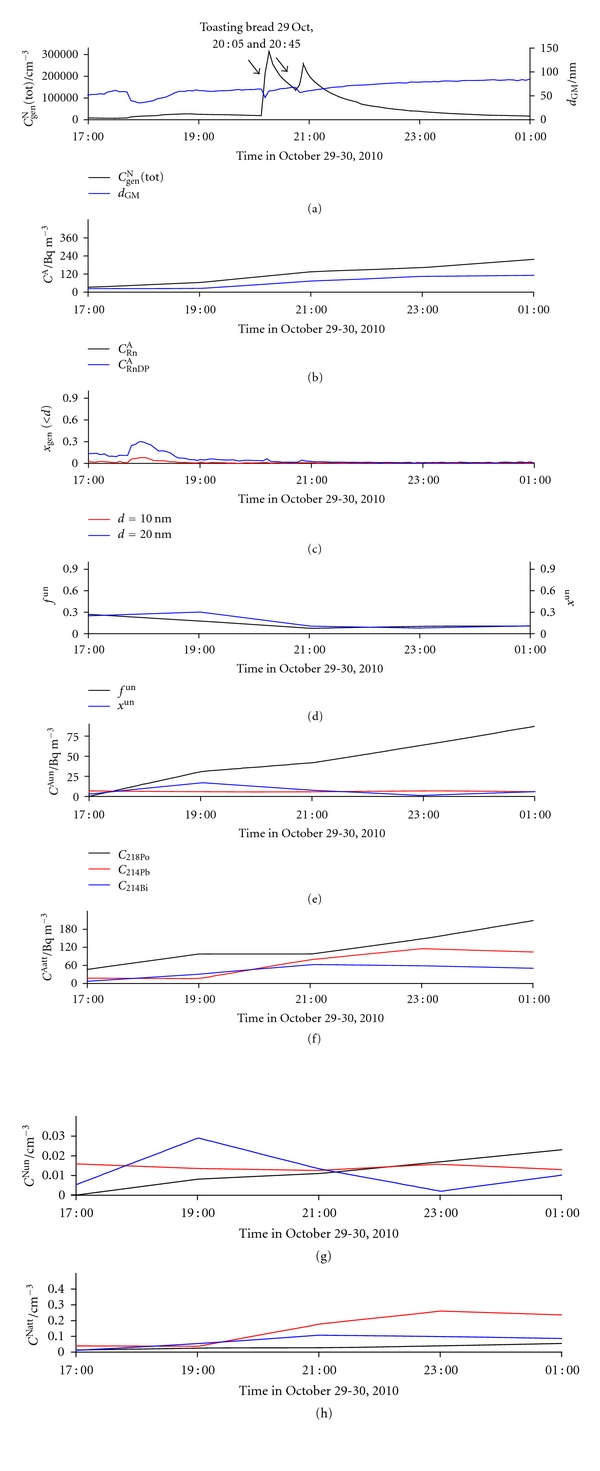
Time run of: (a) total number concentration of general aerosol particles (*C*_gen_^N^(tot)) and geometric mean values of their diameters (*d*_GM_), (b) activity concentration of radon (*C*_Rn_^A^) and equilibrium equivalent activity concentration of radon decay products (*C*_RnDP_^A^), (c) number fraction of particles smaller than 10 nm (*x*_gen_(<10)) and smaller than 20 nm (*x*_gen_(<20)), (d) activity fraction of the unattached RnDP (*f* ^un^) and number fraction of the unattached RnDP (*x* ^un^), (e) activity concentrations of the unattached RnDP atoms (*C*_218_^Aun^_Po_, *C*_214_^Aun^_Pb_, and *C*_214_^Aun^_Bi_), (f) activity concentrations of the attached RnDP atoms (*C*_218_^Aatt^_Po_, *C*_214_^Aatt^_Pb_, and *C*_214_^Aatt^_Bi_), (g) number concentrations of the unattached RnDP atoms (*C*_218_^Nun^_Po_, *C*_214_^Nun^_Pb_, and *C*_214_^Nun^_Bi_), and (h) number concentrations of the attached RnDP atoms (*C*_218_^Natt^_Po_, *C*_214_^Natt^_Pb_, and *C*_214_^Natt^_Bi_), in indoor air for the period October 29-30 (periods of toasting bread in the kitchen from 20:05 to 20:15 and from 20:45 to 20:55 on October 29).

**Figure 16:**
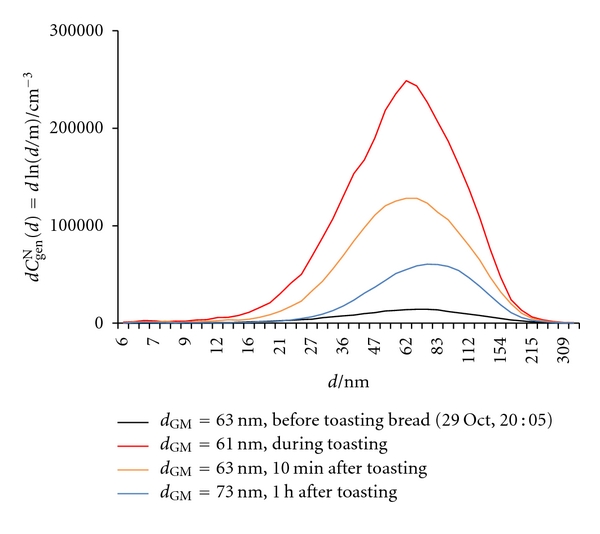
Number size distributions (d*C*_gen_^N^(*d*) = dln(*d*/m)) of general aerosol particles in indoor air in the kitchen during toasting of bread.

An abrupt and very large increase in *C*_gen_^N^(tot) (reaching 300,000 cm^−3^) on October 29 was caused by using a bread toaster (Figure 15(a)). The *C*_gen_^N^(tot) peak is split into two because of two consecutive uses of the toaster for 10 min each, at 20:05 and 20:45. The size distribution of particles did not change significantly during toasting (Figure 16) and was only slightly shifted towards higher sizes afterwards. Decrease in *f* ^un^ and *x* ^un^ is mainly a consequence of decrease in the number concentration of the unattached ^214^Bi (Figure 15(g)) and increase in the number concentration of the attached ^214^Pb (Figure 15(h)). After toasting was finished, *d*_GM_ started to increase continuously, presumably because of particle growth, estimated to be 2-3 nm h^−1^ for urban areas in this season of the year [61, 64].

#### 3.2.4. Cooking a “Risotto” Dish

**Figure 17:**
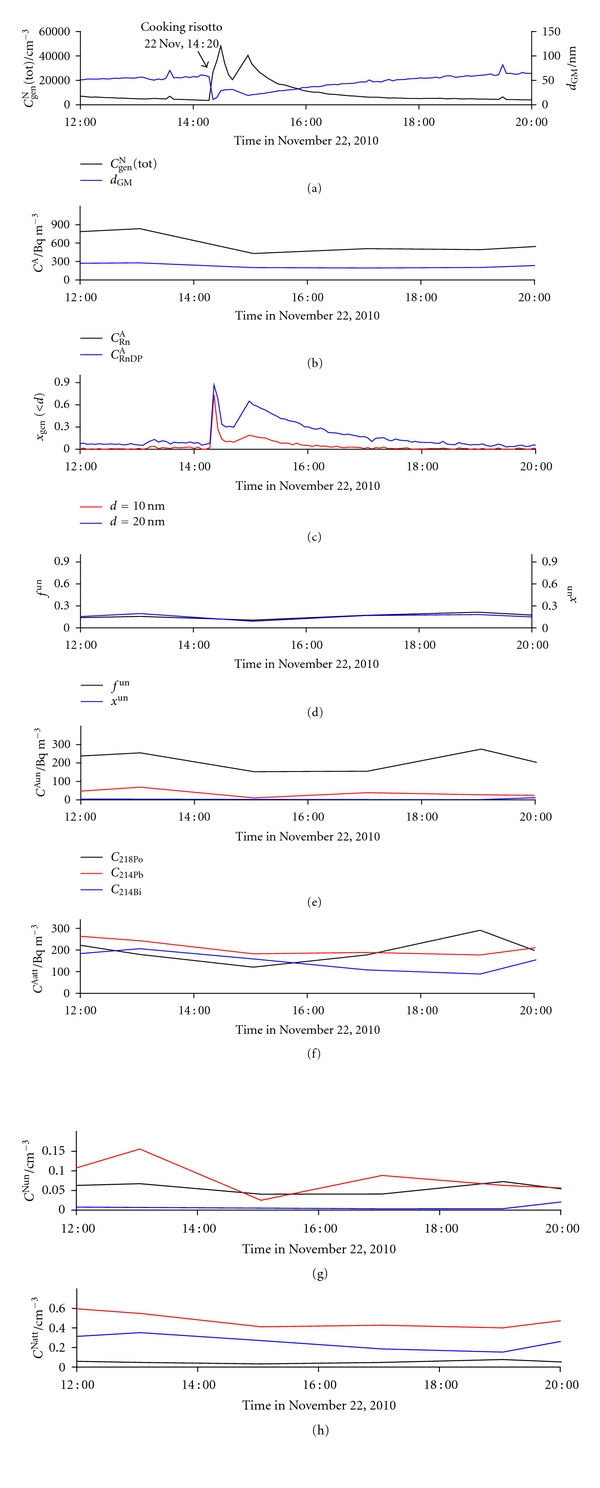
Time run of: (a) total number concentration of general aerosol particles (*C*_gen_^N^(tot)) and geometric mean values of their diameters (*d*_GM_), (b) activity concentration of radon (*C*_Rn_^A^) and equilibrium equivalent activity concentration of radon decay products (*C*_RnDP_^A^), (c) number fraction of particles smaller than 10 nm (*x*_gen_(<10)) and smaller than 20 nm (*x*_gen_(<20)), (d) activity fraction of the unattached RnDP (*f* ^un^) and number fraction of the unattached RnDP (*x* ^un^), (e) activity concentrations of the unattached RnDP atoms (*C*_218_^Aun^_Po_, *C*_214_^Aun^_Pb_, and *C*_214_^Aun^_Bi_), (f) activity concentrations of the attached RnDP atoms (*C*_218_^Aatt^_Po_, *C*_214_^Aatt^_Pb_, and *C*_214_^Aatt^_Bi_), (g) number concentrations of the unattached RnDP atoms (*C*_218_^Nun^_Po_, *C*_214_^Nun^_Pb_, and *C*_214_^Nun^_Bi_), and (h) number concentrations of the attached RnDP atoms (*C*_218_^Natt^_Po_, *C*_214_^Natt^_Pb_, and *C*_214_^Natt^_Bi_), in indoor air on November 22 (cooking a “risotto” dish from 14:20 to 15:00).

**Figure 18:**
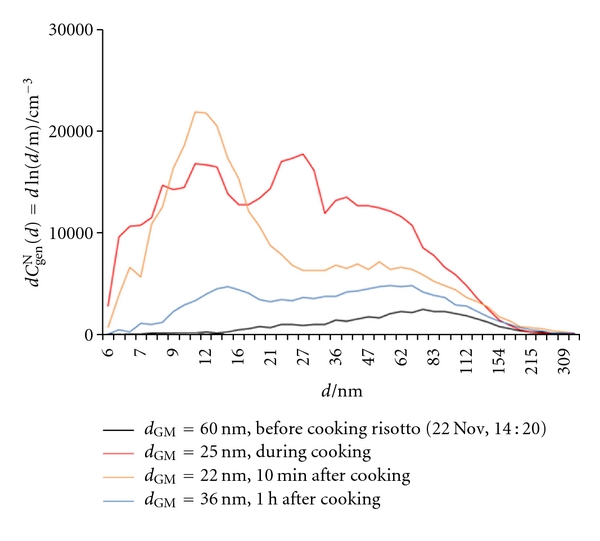
Number size distributions (d*C*_gen_^N^(*d*) = dln(*d*/m)) of general aerosol particles in indoor air in the kitchen during cooking a “risotto” dish.

Figure 17 shows the effect of preparation of an Italian “risotto” dish, including boiling rice and frying rice, vegetables, and additives. *C*_gen_^N^(tot) increased suddenly up to about 50,000 cm^−3^. Particles emitted were of smaller size, thus shifting the size distribution shown in Figure 18 toward the left. More than 80% of the lowered *d*_GM_ was contributed by particles smaller than 20 nm and about 70% by particles smaller than 10 nm (Figure 17(c)). RnDP require some time to be created and then attach to aerosol particles [65]. Therefore, even if the SMPS+C and EQF3020-2 device had the same frequency of analysis, a prompt response of *f* ^un^ and *x* ^un^ to the changes in the general aerosol concentration and size distribution may not be anticipated. The initial conditions were restored more than an hour after the cooking was finished. 

#### 3.2.5. Burning a Candle

**Figure 19:**
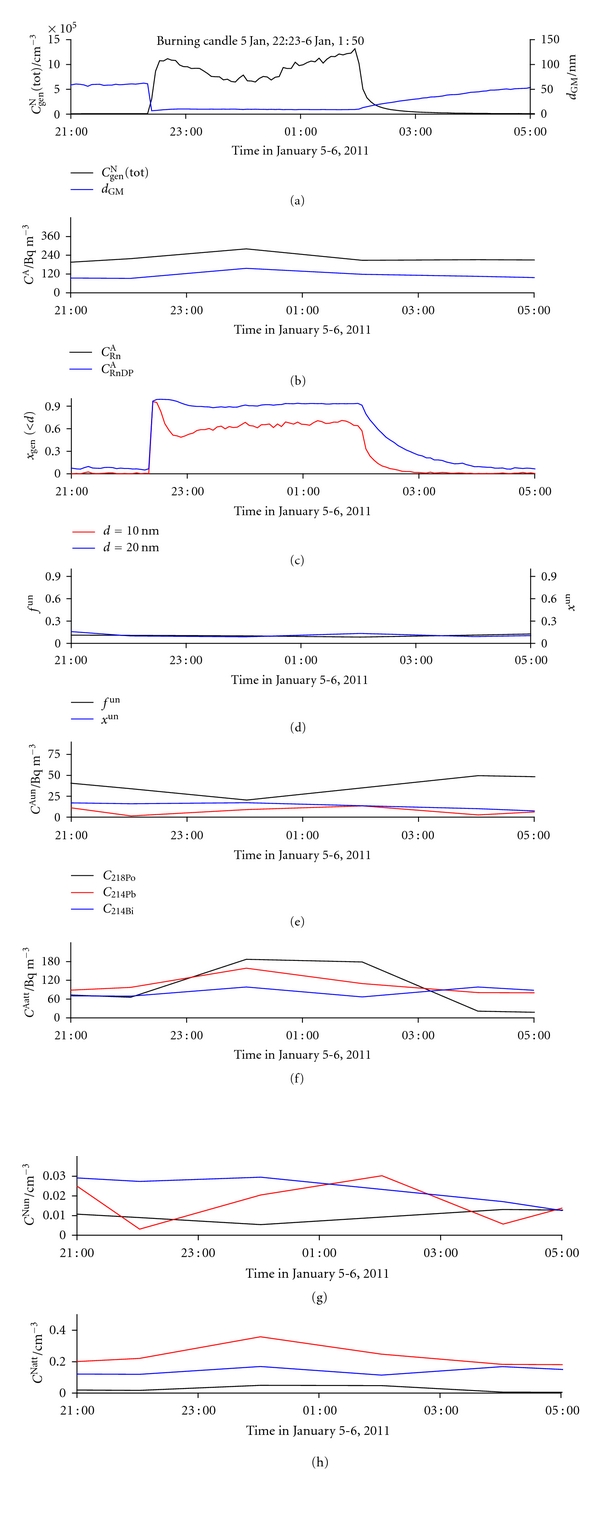
Time run of: (a) total number concentration of general aerosol particles (*C*_gen_^N^(tot)) and geometric mean values of their diameters (*d*_GM_), (b) activity concentration of radon (*C*_Rn_^A^) and equilibrium equivalent activity concentration of radon decay products (*C*_RnDP_^A^), (c) number fraction of particles smaller than 10 nm (*x*_gen_(<10)) and smaller than 20 nm (*x*_gen_(<20)), (d) activity fraction of the unattached RnDP (*f* ^un^) and number fraction of the unattached RnDP (*x* ^un^), (e) activity concentrations of the unattached RnDP atoms (*C*_218_^Aun^_Po_, *C*_214_^Aun^_Pb_, and *C*_214_^Aun^_Bi_), (f) activity concentrations of the attached RnDP atoms (*C*_218_^Aatt^_Po_, *C*_214_^Aatt^_Pb_, and *C*_214_^Aatt^_Bi_), (g) number concentrations of the unattached RnDP atoms (*C*_218_^Nun^_Po_, *C*_214_^Nun^_Pb_, and *C*_214_^Nun^_Bi_), and (h) number concentrations of the attached RnDP atoms (*C*_218_^Natt^_Po_, *C*_214_^Natt^_Pb_, and *C*_214_^Natt^_Bi_), in indoor air for the period January 5-6 (period of burning a candle in the kitchen from 22:23 on January 5 to 1 : 50 on January 6).

**Figure 20:**
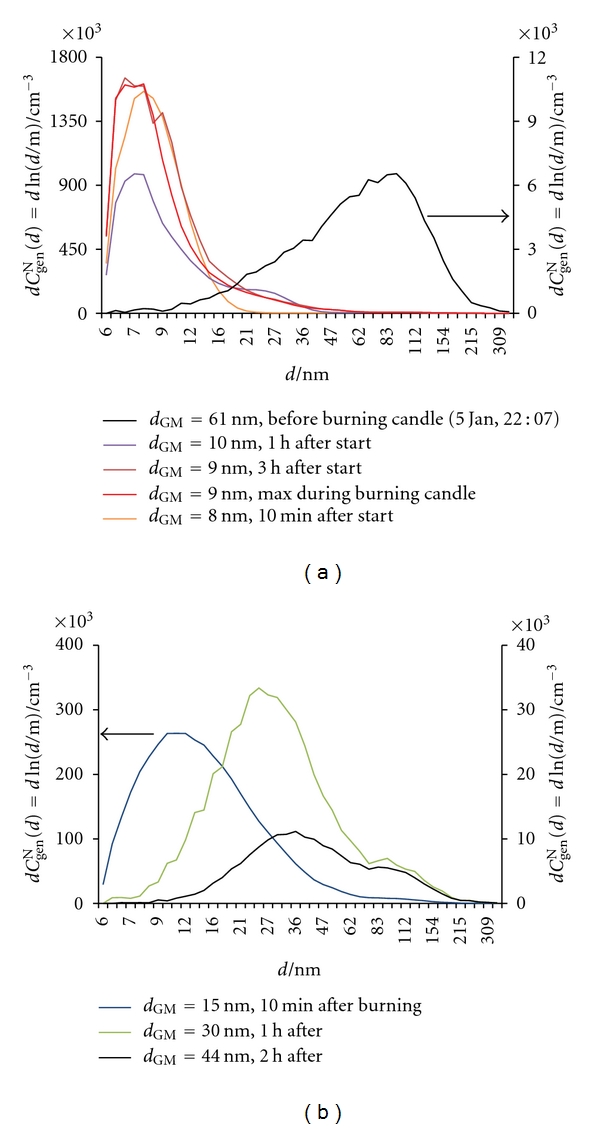
Number size distributions (d*C*_gen_^N^(*d*) = dln(*d*/m)) of general aerosol particles in indoor air (a) before and during burning of a candle and (b) after burning a candle.

An enormous *C*_gen_^N^(tot) peak (reaching 1,320,000 cm^−3^), accompanied by a sudden decrease in *d*_GM_, appeared during burning candle from 22:20 on January 5 to 1 : 50 on January 6 (Figure 19(a)). Particles smaller than 10 nm are produced, as evident from Figure 20. Values of *x*_gen_(<10) and *x*_gen_(<20) exceed 0.60 and 0.90, respectively (Figure 19(c)). This high fraction of small particles should result in high *f* ^un^ values, but it does not. Considerable changes in individual number concentrations of RnDP, in both unattached (Figure 19(e)) and attached form (Figure 19(f)), are compensated in the final result, leaving *f* ^un^ practically unchanged during the entire period (Figure 19(d)), even though an abrupt decrease was expected based on the inverse *f* ^un^–*C*_gen_^N^(tot) relationship [40, 41].

#### 3.2.6. Boiling Water

**Figure 21:**
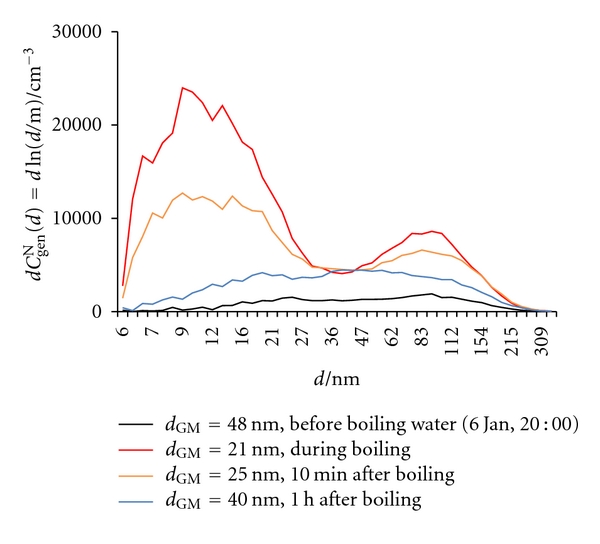
Number size distributions (d*C*_gen_^N^(*d*) = dln(*d*/m)) of general aerosol particles in indoor air before, during, and after boiling water.

**Figure 22:**
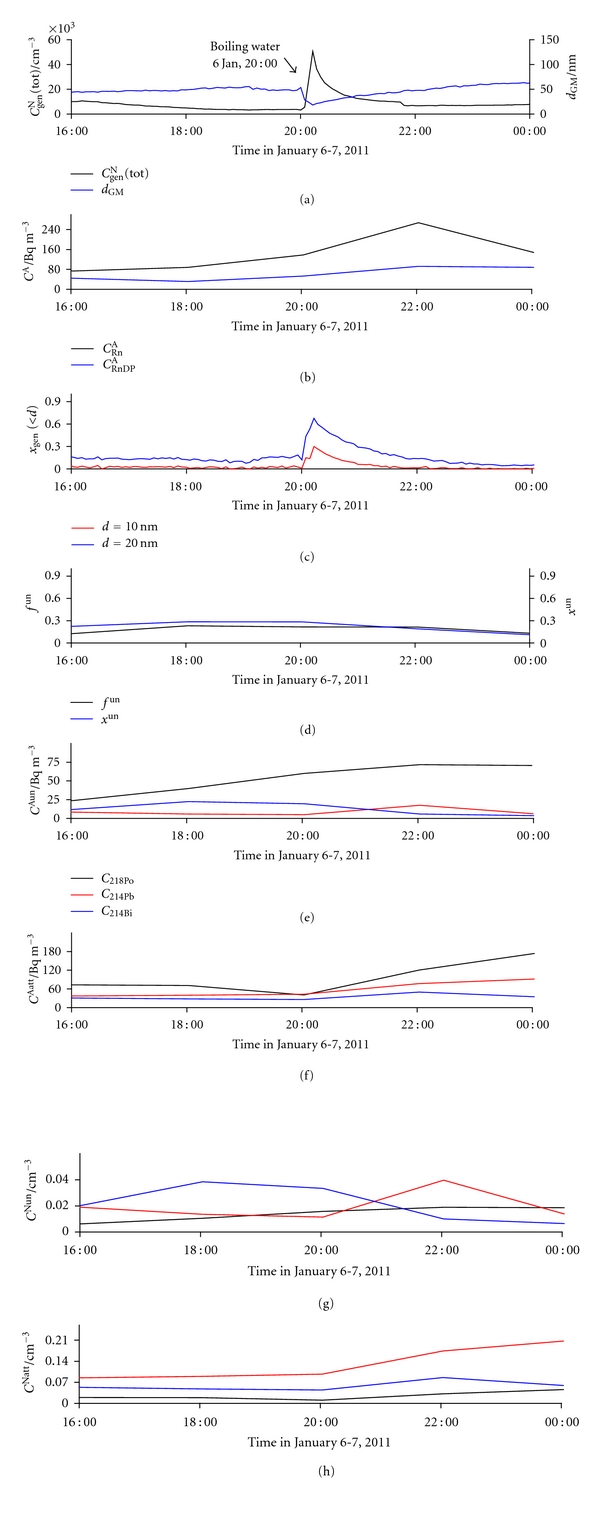
Time run of: (a) total number concentration of general aerosol particles (*C*_gen_^N^(tot)) and geometric mean values of their diameters (*d*_GM_), (b) activity concentration of radon (*C*_Rn_^A^) and equilibrium equivalent activity concentration of radon decay products (*C*_RnDP_^A^), (c) number fraction of particles smaller than 10 nm (*x*_gen_(<10)) and smaller than 20 nm (*x*_gen_(<20)), (d) activity fraction of the unattached RnDP (*f* ^un^) and number fraction of the unattached RnDP (*x* ^un^), (e) activity concentrations of the unattached RnDP atoms (*C*_218_^Aun^_Po_, *C*_214_^Aun^_Pb_, and *C*_214_^Aun^_Bi_), (f) activity concentrations of the attached RnDP atoms (*C*_218_^Aatt^_Po_, *C*_214_^Aatt^_Pb_, and *C*_214_^Aatt^_Bi_), (g) number concentrations of the unattached RnDP atoms (*C*_218_^Nun^_Po_, *C*_214_^Nun^_Pb_, and *C*_214_^Nun^_Bi_), and (h) number concentrations of the attached RnDP atoms (*C*_218_^Natt^_Po_, *C*_214_^Natt^_Pb_, and *C*_214_^Natt^_Bi_), in indoor air on January 6 (boiling water in the kitchen from 20:00 to 20:12).

Before water heating began at 20:00 on January 6, a bimodal, though not well-pronounced, size distribution was observed, with particles grouping at around 25 nm and 85 nm (Figure 21). During heating, water emitted particles smaller than 20 nm, but the contribution of larger particles also grew steadily. Smaller particles are probably clusters of water molecules and larger ones of tiny water droplets. At the boiling point, lasting 10 min, a well pronounced bimodal distribution appeared. After boiling was stopped, a reversed situation was observed. Behaviour of the measured parameters during this process is shown in Figure 22. *C*_gen_^N^(tot) increased suddenly (Figure 22(a)), as did both *x*_gen_(<10) and *x*_gen_(<20), accompanied by a decrease in *d*_GM_ (Figure 22(a)). Because of the small coefficient for ^218^Po in (1), even a large increase in both its number (Figure 22(e)) and activity concentration (Figure 22(g)) during boiling did not contribute enough to increase *f* ^un^ and *x* ^un^, and, hence, they remained practically unchanged (Figure 22(d)).

As already observed during other activities in previous sections, also here the inverse proportionality between *f* ^un^ and *C*_gen_^N^(tot) was not observed.

### 3.3. *f* ^un^–*C*_gen_^N^(tot) Relationship

An inverse proportionality between *f* ^un^ and *C*_gen_^N^(tot) has been observed in a number of cases [40, 41], and Porstendörfer [33] proposed the following empirical relationship for it:

(8) $$f^{\text{un}}=\frac{400}{C_{\text{gen}}^{\text{N}}\left(\text{tot}\right)/\text{c}{\text{m}}^{-3}}.$$

It was also found in the Postojna Cave [40, 45]. In summer, air in the cave is stagnant, concentration of general aerosols is low, and *f* ^un^ is high. The opposite is true in winter, when inflow of fresh air, caused by the chimney effect, introduces outside aerosols, and their concentration in the cave is high, resulting in low *f* ^un^. These are two distinct seasons, separated by weeks or even months. Similarly, higher *f* ^un^ values were observed in flats near parks with lower aerosol concentration than near roads [47].

**Figure 23:**
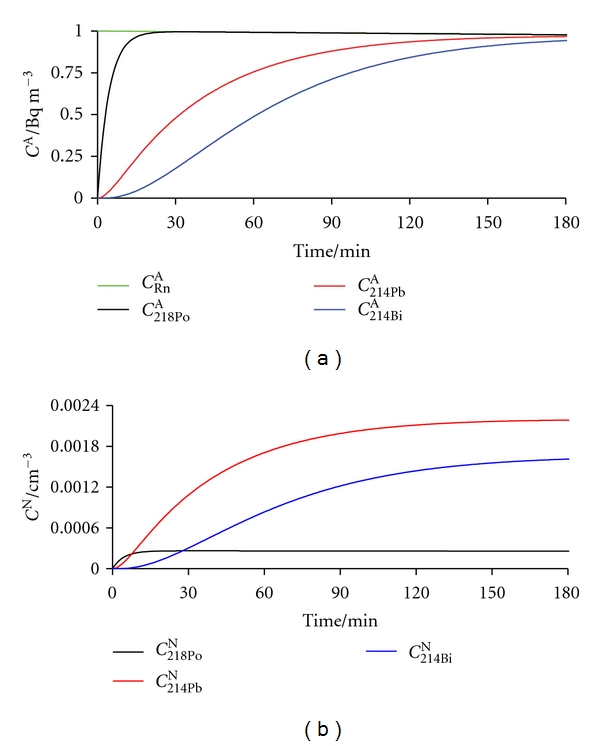
Growth of ^218^Po, ^214^Pb, ^214^Bi, and ^214^Po initiated by *α*-transformation of ^222^Rn (initial activity concentration of 1 Bq m^−3^, equivalent to 0.477 radon atoms cm^−3^), expressed in: (a) activity concentration and (b) number concentration of RnDP atoms.

In our study, such proportionality was not observed. Changes in number concentration and size distribution of general aerosols were fast, lasting only minutes, or at most two hours (in the case of burning a candle). It is not expected that a change in size distribution will cause an immediate redistribution between unattached and attached RnDP. It will rather influence only the newly born RnDP atoms and clusters. Creation of RnDP atoms by radioactive transformations takes time (Figure 23); also, their neutralization, clustering, and attachment to [65] and detachment from general aerosol particles by recoil are processes with defined values of rate constants. Taking this into account, calculation would show [66] that a time delay of even more than hours [67] necessarily appears between a change in particle size distribution of general aerosols and a change in *f* ^un^. Therefore, a true dependence of *f* ^un^ on the changes in general aerosols in our study was masked or even totally obscured. We speculate that most probably this is the main reason for our failure to observe the expected inverse proportionality, and not only the considerable difference in the analysis frequencies of the two devices (once every 4 minutes for SMPS+C and once in two hours for EQF3020-2). Nevertheless, it would be useful to repeat some measurements using the devices with similar analysis frequencies.

**Figure 24:**
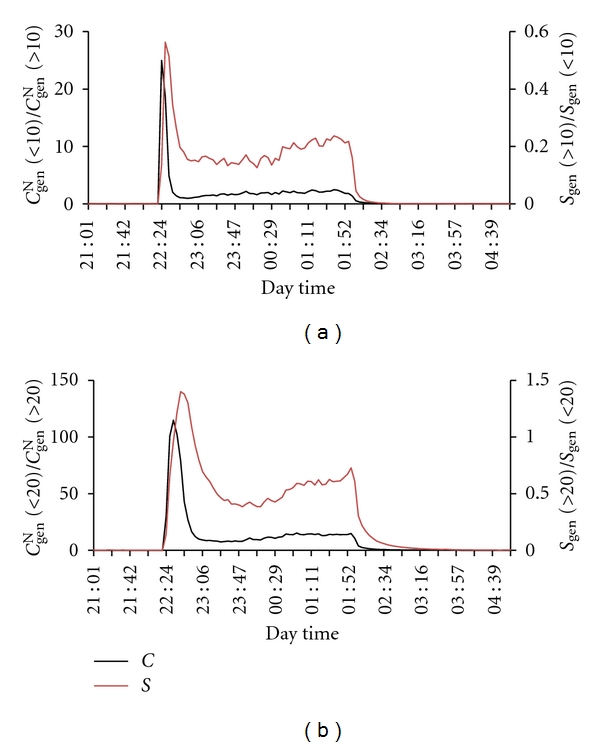
Conditions of general aerosols during burning of a candle on January 5-6: (a) ratio of the number concentration of aerosol particles smaller than 10 nm (*C*_gen_^N^(<10)) to that of bigger ones (*C*_gen_^N^(>10)) and ratio of the total surface area of all particles smaller than 10 nm (*S*_gen_(<10)) to the total surface area of all particles bigger than 10 nm (*S*_gen_(>10)) in a volume unit and (b) ratio of the number concentration of aerosol particles smaller than 20 nm (*C*_gen_^N^(<20)) to that of bigger ones (*C*_gen_^N^(>20)) and ratio of the total surface area of all particles smaller than 20 nm (*S*_gen_(<20)) to the total surface area of all particles bigger than 20 nm (*S*_gen_(>20)) in a volume unit.

Another reason may be also found in Figure 24, in which ratios of the number concentrations and total surface area of the smaller and larger particles are plotted for the period during candle burning. *C*_gen_^N^(<10) is about 2-fold higher than *C*_gen_^N^(>10), and the total surface area of smaller particles in a volume unit (*S*_gen_^N^(<10)) is about 5-fold lower than that of the larger ones (*S*_gen_^N^(>10)) (Figure 24(a)). In the case of <20 nm particles, the situation is as follows (Figure 24(b)): *C*_gen_^N^(<20) is about 10-fold higher than *C*_gen_^N^(>20), and the total surface area of smaller particles (*S*_gen_^N^(<20)) is about 2-fold lower than that of the larger ones (*S*_gen_^N^(>20)). Therefore, a preference of RnDP atoms (clusters) for association with particles smaller than 20 nm and smaller than 10 nm, with resulting lower *f* ^un^ values, may not be expected. It may be concluded that it is the total surface area of the smaller particles that controls the *f* ^un^ value and neither the total number aerosol concentration nor the number concentration of smaller particles.

## 4. Conclusion

Radon decay products (RnDP) and general aerosols were monitored simultaneously in a basement kitchen and in the courtyard of a farm in a suburban area. In the outdoor air, the total number concentration (*C*_gen_^N^(tot)) of general aerosols varies from 1000 cm^−3^ to 86,000 cm^−3^, with two daily maxima, one at around midnight and the other between 8:00 and 16:00. Number size distribution did not show a regular diurnal variation but rather varied from day to day, depending mostly on the solar radiation and intensity of farming equipment use.

In the indoor air, during periods without any human activity and with the window closed, *C*_gen_^N^(tot) varied from 2000 to 13,000 cm^−3^, *d*_GM_ from 22 to 87 nm, *C*_Rn_^A^ from 28 to 834 Bq m^−3^, *C*_RnDP_^A^ from 24 to 278 Bq m^−3^, and *f* ^un^ from 0.09 to 0.28. *C*_gen_^N^(tot) increased substantially during toasting of bread and burning of a candle, reaching 670,000 cm^−3^ and 1,320,000 cm^−3^, respectively.

Even these large changes in concentration in indoor air did not change *f* ^un^ significantly, and the inverse proportionality of the *f* ^un^–*C*_gen_^N^(tot) relationship was not observed. Thus, even considerable effects of human activity on the general aerosol conditions were not reflected in changes of *f* ^un^, the important parameter in radon dosimetry. Obviously, not only the number concentration of general aerosols affects *f* ^un^ but also the particle size distribution. Although during the candle burning the concentration of particles smaller than 10 nm was several times higher than that of larger ones, in contrast, their total surface area was several times lower than that of the larger ones. Therefore, an enhancement of preferential association of RnDP atoms or clusters to smaller particles of general aerosols, and thus increasing *f* ^un^, can hardly be expected. In addition, creation of RnDP by radioactive transformation and their neutralization, clustering, and association with general aerosol particles are processes with certain values of rate constants. Therefore, the response of *f* ^un^ to the changes in general aerosols is necessarily delayed, and a correct *f* ^un^–*C*_gen_^N^(tot) relationship is obscured.

## Figures and Tables

**Table 1:** Minimum and maximum values, geometric means, and geometric standard deviations (except for *R*_*s*_ for which arithmetic mean and arithmetic standard deviation are given) for the total number concentration of general aerosol (*C*_gen_^*N*^ (tot)), geometric mean of number size distribution of general aerosol (*d*_GM_), number fractions of particles smaller than 10 nm (*x*_gen_(<10)) and smaller than 20 nm (*x*_gen_(<20)), equilibrium equivalent concentration of radon short-lived decay products (*C*_RnDP_^*A*^), activity fraction of the unattached radon short-lived decay products (*f* ^un^), barometric pressure (*P*), air temperature (*T*), air relative humidity (*H*_*rel*⁡_), wind speed (*v*_*w*_), solar radiation (*R*_*s*_), and concentrations of aerosol particles smaller than 10 *μ*m (*C*_PM10_), SO_2_ (*C*_SO_2__), NO_x_ (*C*_NO_*x*__), and O_3_ (*C*_O_3__) for the entire period of measurements outdoors in May (3 May–6 June) and October (7–11 October, 17–21 October, and 24–26 October).

Parameter	May				October			
	min	max	GM	GSD	min	max	GM	GSD

*C*_gen_^N^(tot)/cm^−3^	1420	75650	6830	1.69	970	85930	6940	1.93
*d*_GM_/nm	18	124	53	1.42	15	133	59	1.43
*x*_gen_ (<10)					0.03	0.37	0.02	3.08
*x*_gen_ (<20)	0.03	0.78	0.12	2.47	0.03	0.79	0.09	2.75
*C*_RnDP_^A^/Bq m^−3^					2	18	6	2.05
*f* ^un^					0.01	0.75	0.09	2.38
*P*/hPa	963	988	977	1.01	970	993	981	1.01
*T*/°C	5	28	15	1.31	1	16	8	1.56
*H*_*rel*⁡_/%	21	99	65	1.43	42	99	82	1.19
*v*_w_/m s^−1^	0.1	7	1.2	3.16	0.1	5	0.8	2.33
*R*_*s*_/W m^−2^	1	1085	198*	266**	1	793	78*	145**
*C*_PM10_/*μ*g m^−3^	0.03	52	13	3.22	0.03	113	23	3.26
*C*_SO_2__/*μ*g m^−3^	0.1	8	0.7	3.40	0.1	14	0.6	3.87
*C*_NO_*x*__/*μ*g m^−3^	4	129	24	1.97	4	209	38	2.14
*C*_O_3__/*μ*g m^−3^	1	159	42	2.60	1	92	14	3.22

*Arithmetic mean.

**Arithmetic standard deviation.

**Table 2:** Minimum and maximum values, geometric means, and geometric standard deviations for the total number concentration of general aerosol (*C*_gen_^*N*^(tot)), geometric mean of number size distribution of general aerosol (*d*_GM_), number fractions of particles smaller than 10 nm (*x*_gen_(<10)) and smaller than 20 nm (*x*_gen_(<20)), radon activity concentration (*C*_Rn_^*A*^), equilibrium equivalent concentration of radon short-lived decay products (*C*_RnDP_^*A*^), equilibrium factor between radon and radon short-lived decay products (*F*), activity fraction of the unattached radon short-lived decay products (*f* ^un^), and number fraction of the unattached radon short-lived decay products (*x* ^un^), for the entire period of measurements indoors (28–31 October, 21-22 November, and 5-6 January), only when the door and window were closed and without any human activity.

Parameter	28 October–6 January			
	min	max	GM	GSD

*C*_gen_^N^(tot)/cm^−3^	1840	13260	5120	1.50
*d*_GM_/nm	22	87	57	1.24
*x*_gen_(<10)	0.001	0.30	0.01	2.63
*x*_gen_(<20)	0.004	0.59	0.08	2.08
*C*_Rn_^A^/Bq m^−3^	28	834	229	1.96
*C*_RnDP_^A^/Bq m^−3^	24	278	101	1.85
*F*	0.24	0.67	0.43	1.24
*f* ^ un^	0.09	0.28	0.16	1.30
*x* ^ un^	0.08	0.36	0.17	1.45
